# Digital food environments, food literacy, and sustainable eating among children in China: evidence from National Panel Data on supermarket availability and nutritional status

**DOI:** 10.3389/fnut.2026.1908801

**Published:** 2026-08-19

**Authors:** Bin Liang, Liang Tian

**Affiliations:** Department of Paediatrics, Sichuan Provincial People’s Hospital, School of Medicine, University of Electronic Science and Technology of China, Chengdu, Sichuan, China

**Keywords:** child nutritional status, China, digital food environment, food literacy, supermarket availability, sustainable eating

## Abstract

Children’s dietary behavior in China is increasingly shaped by digital food environments, yet how digital exposure interacts with physical food access and food literacy in relation to child nutrition remains unclear at a national scale. This study utilized a longitudinal province-year panel covering 31 mainland Chinese provinces from 2006 to 2025 (*N* = 620). Core variables—specifically the health-promoting and risk-enhancing digital food environments, food literacy, supermarket availability, and sustainable eating—were operationalized as macro-level indices using the entropy weighting method based on regional statistical and household consumption data. To evaluate these constructs against child nutritional outcomes, we estimated two-way fixed-effects models, mediation and moderation pathways, and geographically weighted regressions. Health-promoting digital food environments were positively associated with sustainable eating (*β* = 0.115), with food literacy mediating 21.8% of the total association. Supermarket availability strengthened this beneficial association (*β* = 0.073), while food literacy buffered the risk-enhancing digital pathway toward unhealthy eating tendencies (*β* = −0.087). In lagged models, sustainable eating was associated with lower child nutritional risk (*β* = −0.124). Regional heterogeneity indicated that digital pathways were stronger in more developed provinces, whereas physical food retail availability mattered more in western regions. These findings support integrated, region-sensitive strategies that combine digital health access, food literacy promotion, and physical retail development.

## 1 Introduction

Child nutrition in China has changed markedly over the past two decades. Although undernutrition has declined, overweight, obesity, poor dietary diversity, and unequal access to high-quality foods have become increasingly important public-health concerns (1, 2). These shifts have occurred alongside rapid urbanization, growth of modern food retail, rising incomes, and the platformization of food consumption (3). Consequently, children’s diets are shaped not only by household socioeconomic conditions and neighborhood food outlets but also by the interaction between digital food environments and physical food access (4).

Digital food environments now influence how families search for, purchase, and evaluate food through online retail, delivery platforms, short-video and livestream marketing, digital payments, and health-information services (5, 6). In China, digital exposure is closely linked to food access, food marketing, and obesity-related risk (7, 8). Yet digitalization is not uniformly beneficial or harmful. Health-promoting resources may expand access to nutrition information, fresh-food services, and digital health support, whereas risk-enhancing platforms can intensify exposure to fast food, sugar-sweetened beverages, snacks, and processed foods (9). Evidence that unhealthy foods are more visible online than healthy options further suggests that these pathways can operate simultaneously (10).

Physical food access can condition whether digital opportunities translate into dietary improvement. Supermarkets provide a broad range of foods and may help households act on nutrition knowledge and online health information (11, 12). Where access is limited, however, digital platforms may reinforce convenience-oriented consumption and dependence on takeaway or processed foods (13). Digital and physical food environments should therefore be considered jointly (14). Chinese evidence also associates better supermarket access with greater dietary diversity and nutrient intake among children, particularly in rural and lower-income settings (15).

Food literacy provides a plausible mechanism linking these environments to behavior. It encompasses the ability to interpret nutrition information, evaluate food quality and labels, manage food choices, and make health- and sustainability-oriented decisions (16–18). In the health-promoting pathway, food literacy may translate digital information and fresh-food access into sustainable dietary practices, thereby acting as a mediator (19). In the risk-enhancing pathway, it may help families recognize marketing and resist promotions for high-sugar, high-fat, and highly processed foods, thereby buffering adverse exposure (20). This asymmetric role allows the same cognitive capacity to facilitate beneficial resources while limiting environmental risk.

Sustainable eating is positioned as the behavioral link between food environments and child nutritional outcomes (21). Here it refers to a balanced, diverse, health-oriented dietary pattern that is broadly consistent with long-term food-system sustainability (22, 23). Sustainable eating and unhealthy eating tendencies are treated as related but distinct dimensions rather than opposite ends of a single continuum. This distinction reflects China’s dietary transition, in which traditional healthy foods and commercially promoted ultra-processed foods may coexist within the same regional food system (24). It also permits the health-promoting and risk-enhancing digital pathways to be evaluated separately.

Despite growing work on digital food exposure, supermarket access, and food literacy, three gaps remain. First, these factors are often examined separately rather than within an integrated digital–physical framework (25). Second, studies rarely distinguish health-promoting from risk-enhancing digital environments or test how food literacy and supermarket availability modify their associations with diet. Third, much of the evidence is cross-sectional or city-specific, limiting assessment of long-term regional change (26). Accordingly, this study examines how the two digital food environment dimensions are associated with sustainable and unhealthy eating; whether food literacy mediates or buffers these relationships; whether supermarket availability strengthens the health-promoting pathway; and whether dietary patterns are associated with subsequent child nutritional outcomes. We construct a province-year panel covering 31 mainland Chinese provinces from 2006 to 2025 and apply two-way fixed-effects, mediation, moderation, and spatial models. By integrating digital exposure, physical food access, and food literacy, the study provides longitudinal evidence on regionally heterogeneous nutrition pathways in China.

## 2 Methods

### 2.1 Study design

This study adopted a province-level longitudinal panel design to examine the relationships among digital food environments, supermarket availability, food literacy, sustainable eating, and child nutritional status in China. The analytical unit was the province-year. The study covered 31 mainland Chinese provinces, autonomous regions, and municipalities from 2006 to 2025. Maximum sample size was 620 province-year observations, but nutritional outcome models used smaller available-year subpanels where child health indicators were not available annually.

Province-level panel data were used rather than national aggregate time-series data because 20 annual national observations would not be sufficient for fixed-effects estimation, mediation analysis, moderation testing, and heterogeneity analysis. The province-year panel structure allows the study to capture both temporal changes and cross-regional differences in China’s food environment transition. Because the design is observational and reliant on aggregated province-level panels, the estimates must be strictly interpreted as macro-level ecological associations rather than individual-level causal effects. To explicitly avoid the ecological fallacy, all inferences regarding dietary behavior and nutritional status in this study are bounded to population-level trends and structural environmental exposures, rather than individual psychological or behavioral mechanisms.

### 2.2 Data sources

Data were compiled from China Statistical Yearbooks, provincial statistical yearbooks, China Health Statistical Yearbooks, China Education Statistical Yearbooks, China Commerce Yearbooks, household consumption statistics, food price statistics, internet development reports, and available child health or student physical fitness monitoring data. All indicators were harmonized by province and year.

Given the standard publication lag of official yearbooks, complete provincial statistical yearbooks for 2025 were not fully available at the time of initial data compilation. To address this, 2025 observations were rigorously supplemented using official provincial statistical communiqués and verified sectoral reports released in early 2026. For any specific indicators where 2025 finalized figures remained formally unpublished, the latest available data from 2024 were utilized to maintain empirical integrity, ensuring that no artificial or unverified values were forced into the panel. To ensure measurement transparency and address missing data rigorously, we applied distinct missing-data protocols depending on the variable type. For independent and intermediate variables (e.g., digital environments, food literacy, and supermarket availability), occasional missing intermediate-year observations were handled via linear interpolation to maintain the longitudinal continuity of the province-year panel. Conversely, for the dependent child nutritional outcomes, no unobserved values were imputed. Missing nutritional endpoint data were strictly handled via listwise deletion, resulting in a naturally unbalanced subpanel for outcome estimations. This conservative protocol prevents the introduction of artificial variance or estimation bias into the final health endpoints.

### 2.3 Conceptual and analytical framework

Drawing on the socio-ecological model of health behavior, this study conceptualizes child dietary and nutritional outcomes as products of interconnected digital, physical, and cognitive contexts. The analytical framework comprises two parallel exposure pathways: health-promoting digital food environments are examined in relation to sustainable eating, whereas risk-enhancing digital food environments are examined in relation to unhealthy eating tendencies; unhealthy eating tendency is then evaluated in relation to nutritional risk.

The framework also evaluates three mechanisms. Food literacy is examined as a mediator between health-promoting digital food environments and sustainable eating and as a moderator of the risk-enhancing pathway. Supermarket availability is examined as a moderator of the health-promoting pathway. Finally, lagged sustainable eating is evaluated in relation to child nutritional status and nutritional risk. All specifications are interpreted as province-level associations rather than individual-level causal effects.

### 2.4 Health-promoting digital food environment

The health-promoting digital food environment (HP-DFE) captures the foundational structural capacity and digital inclusion necessary for health-enhancing dietary behaviors. Rather than measuring direct individual exposure to specific healthy foods, these macro-level indicators—such as internet penetration, broadband access, digital payment capacity, online fresh food access, and digital health service use—proxy the prerequisite regional infrastructure required for households to seek nutrition information and utilize modern, formalized fresh food supply chains. While foundational technologies (e.g., broadband and digital payment) are inherently neutral and can facilitate multiple types of consumption, they are theoretically classified here as structural prerequisites for digital health literacy and formal food access. A higher value indicates a structurally enabling environment that empowers positive dietary decision-making.

### 2.5 Risk-enhancing digital food environment

Conversely, the risk-enhancing digital food environment (RE-DFE) specifically captures structural exposure to commercialized, convenience-oriented digital food marketing. It includes indicators such as online food delivery penetration, platform-based catering consumption, short-video and livestream marketing exposure, and fast-food/processed food consumption. To address potential discriminant validity concerns between the dual pathways, our classification theoretically distinguishes between foundational digital infrastructure (HP-DFE) and specific commercial market applications (RE-DFE). We acknowledge that catering or short-video platforms do not exclusively promote unhealthy foods; however, at the aggregate provincial level, the rapid expansion of these specific commercial channels is disproportionately driven by algorithmic marketing of energy-dense foods and convenience-based options. Thus, while HP-DFE captures the capacity for healthy choices, RE-DFE isolates the intensive commercial ecosystem that structurally incentivizes unhealthy eating tendencies.

When direct province-level food delivery data were unavailable, provincial food delivery exposure was proxied by combining national online food delivery penetration with provincial digital access and catering consumption intensity:

$$FD_{\mathrm{pt}}={\text{NFOD}}_{\mathrm{pt}}\cdot \text{Interne}t_{\mathrm{pt}}\cdot \text{Caterin}g_{\mathrm{pt}}$$

where $FD_{\mathrm{pt}}\;$ denotes provincial food delivery exposure, NFOD_t denotes national online food delivery penetration in year t, $\text{Interne}t_{\mathrm{pt}}$ denotes provincial internet penetration, and $\text{Caterin}g_{\mathrm{pt}}$ denotes per capita catering expenditure or catering revenue.

### 2.6 Food literacy

In this province-level study, food literacy ( $FL_{\mathrm{pt}}$ ) is operationalized not as an individual psychological trait, but as the regional ecological capacity that supports household food decision-making. Because children’s diets are primarily governed by parental and caregiver choices, this macro-level proxy inherently captures the aggregate human capital and health awareness of adults within the household. We acknowledge the conceptual distinction between general cognitive ability and specific nutritional knowledge; therefore, our composite proxy mitigates this gap by integrating foundational cognitive indicators (e.g., average years of schooling) with domain-specific measures (e.g., regional health literacy rates, nutrition education resources, and school health education resources). While this ecological operationalization cannot fully capture individual-level heterogeneity in digital engagement, it serves as a robust macro-level proxy for the structural capacity required to buffer digital food marketing risks and act upon health-promoting information at the population level.

Average years of schooling were calculated using weighted educational attainment:

$${\text{EduYear}}_{\mathrm{pt}}=\frac{6\mathrm{Pr}\text{imar}y_{\mathrm{pt}}+9\text{Junio}r_{\mathrm{pt}}+12\text{Senio}r_{\mathrm{pt}}+16\text{Colleg}e_{\mathrm{pt}}}{\text{Populatio}n_{\mathrm{pt}}}$$

where $\mathrm{Pr}\text{imar}y_{\mathrm{pt}}$ , $\text{Junio}r_{\mathrm{pt}}$ , $\text{Senio}r_{\mathrm{pt}}$ and $\text{Colleg}e_{\mathrm{pt}}$ represent the number of people with primary, junior secondary, senior secondary, and college-or-above education in province p and year t. A higher value indicated stronger regional human capital and potential food literacy.

### 2.7 Supermarket availability

Supermarket availability, denoted as $SA_{\mathrm{pt}}$ , measured regional access to modern food retail resources. It included the number of supermarkets, density of retail enterprises, supermarket sales, food retail sales, supermarket business area, and per capita food retail resources.

Because the study used province-level data, supermarket availability was defined as regional supermarket availability rather than individual distance to the nearest supermarket. Core indicators were calculated as:

$${\text{SupermarketDensity}}_{\mathrm{pt}}=\frac{\text{Supermarket}s_{\mathrm{pt}}}{{\text{Population}}_{\mathrm{pt}}}\cdot 100000$$

RetailIntensitypt=FoodRetailSalesptPopulationpt

A higher value of $SA_{\mathrm{pt}}$ indicated better physical access to diversified food resources.

### 2.8 Unhealthy eating tendency

Unhealthy eating tendency, denoted as $UE_{\mathrm{pt}}$ , measured regional dietary patterns associated with nutritional risk. It was constructed from sugar-sweetened beverage consumption, processed food consumption, fried food consumption, catering expenditure, excessive edible oil consumption, excessive sugar consumption, excessive red meat consumption, and insufficient fruit and vegetable intake.

Positive risk indicators were coded so that higher values represented stronger unhealthy eating tendencies. For insufficient intake of healthy foods, reverse coding was applied. The final $UE_{\mathrm{pt}}$ index reflected a regional tendency toward high-sugar, high-fat, high-salt, processed, and convenience-based dietary patterns.

### 2.9 Sustainable eating

The sustainable eating index, denoted as $\mathrm{SE}I_{\mathrm{pt}}$ , measured the extent to which provincial dietary patterns aligned with the FAO/WHO framework for ‘Sustainable Healthy Diets.’ While data constraints preclude the direct inclusion of ecological footprint metrics (e.g., greenhouse gas emissions), this macro-index captures the nutritional and structural dimensions of sustainability. By emphasizing a higher plant-based food proportion alongside balanced animal-source food intake, the index inherently reflects dietary patterns that exert lower environmental impacts while optimizing human health. Positive components included vegetable intake, fruit intake, dairy intake, legume and soy product intake, appropriate grain consumption, plant-based food proportion, and balanced animal-source food intake.

Risk components, including excessive red meat intake, edible oil consumption, sugar-sweetened beverage consumption, and processed food consumption, were reverse-coded. The index was calculated as:

$$\mathrm{SE}I_{\mathrm{pt}}=\sum_{j=1}^{m}w_{j}Z_{\mathrm{jpt}}^{+}+\sum_{k=1}^{n}w_{k}Z_{\mathrm{kpt}}^{-}$$

where $Z_{\mathrm{jpt}}^{+}$ denotes standardized positive dietary indicators and $Z_{\mathrm{kpt}}^{-}$ denotes reverse-coded risk dietary indicators.

### 2.10 Child nutritional status and nutritional risk

Child nutritional status, denoted as $CN_{\mathrm{pt}}$ , was the final health outcome. To ensure epidemiological reliability, the raw anthropometric and clinical data for these endpoints were systematically extracted from official provincial health statistical yearbooks, the Chinese National Surveys on Students’ Constitution and Health (CNSSCH), and periodically published national child nutrition monitoring reports. The composite outcome included child and adolescent BMI-related indicators, height-related indicators, overweight rate, obesity rate, thinness rate, stunting or growth retardation rate, anemia rate, and other available nutrition-related indicators.

Nutritional risk, denoted as $NR_{\mathrm{pt}}$ , was constructed as the negative dimension of child nutritional status. It included overweight, obesity, thinness, stunting, anemia, and other adverse nutrition-related indicators:

$$NR_{\mathrm{pt}}=\sum_{r=1}^{R}w_{r}Z_{\mathrm{rpt}}$$

A higher value of $NR_{\mathrm{pt}}$ indicated greater nutritional risk. If annual province-level child nutritional indicators were not fully available, the nutritional outcome models were estimated using the available province-year subpanel.

### 2.11 Standardization and index construction

To ensure strict measurement transparency and prevent endogeneity driven by definitional overlap, we meticulously separated digital exposure indicators from dietary behavior indicators during index construction. Variables capturing environmental infrastructure and market presence (used for HP-DFE and RE-DFE) were mutually exclusive from variables capturing actual household food consumption volumes (used for UE and SEI). No single proxy was shared across the exposure and behavioral constructs.

Following this strict separation, all raw indicators were standardized before index integration. For positive indicators, the following min–max transformation was used:

$$Z_{\mathrm{jpt}}=\frac{X_{\mathrm{jpt}}-\min(X_{j})}{\max(X_{j})-\min(X_{j})}$$

For negative indicators, the transformation was:

$$Z_{\mathrm{jpt}}=\frac{\max(X_{j})-X_{\mathrm{jpt}}}{\max(X_{j})-\min(X_{j})}$$

where $X_{\mathrm{jpt}}$ is the raw value of indicator j in province p and year t, and $Z_{\mathrm{jpt}}$ is the standardized value. The main analysis used the entropy weighting method:

$$P_{\mathrm{jpt}}=\frac{Z_{\mathrm{jpt}}}{\sum Z_{\mathrm{jpt}}}$$

$$e_{j}=-k\sum P_{\mathrm{jpt}}\ln(P_{\mathrm{jpt}})$$

$$w_{j}=\frac{1-e_{j}}{\sum (1-e_{j})}$$

$$\text{Inde}x_{\mathrm{pt}}=\sum w_{j}Z_{\mathrm{jpt}}$$

Equal-weighted indices and principal component analysis-based indices were used in robustness checks.

### 2.12 Control variables

The models controlled for socioeconomic, demographic, educational, health, and price-related provincial characteristics. These included per capita GDP, urbanization rate, per capita disposable income, household consumption level, child population proportion, education expenditure, public health expenditure, number of physicians per 1,000 population, number of health institutions, food consumer price index, vegetable price index, meat price index, and fruit price index.

A COVID-19 period dummy variable was also included because 2020–2022 may have changed online food purchasing, food delivery use, household food access, children’s physical activity, and dietary behavior:

$${\text{COVID}}_{t}\{\begin{array}{c} 1,2020\leq t\leq 2022\\ 0,\text{otherwise} \end{array}$$

### 2.13 Baseline fixed-effects model

Two-way fixed-effects models were used to control for time-invariant provincial characteristics and common year shocks. The general specification was:

$$Y_{\mathrm{pt}}=\beta_{0}+\beta_{1}\mathrm{DF}E_{\mathrm{pt}}+\beta_{2}SA_{\mathrm{pt}}+\beta_{3}FL_{\mathrm{pt}}+\gamma X_{\mathrm{pt}}+\mu_{p}+\lambda_{t}+\varepsilon_{\mathrm{pt}}$$

where $Y_{\mathrm{pt}}$ denotes the outcome variable, $\mathrm{DF}E_{\mathrm{pt}}$ denotes the digital food environment variable, $SA_{\mathrm{pt}}$ denotes supermarket availability, FL_pt denotes food literacy, $X_{\mathrm{pt}}$ denotes control variables, $\mu_{p}$ denotes province fixed effects, $\lambda_{t}$ denotes year fixed effects, and $\varepsilon_{\mathrm{pt}}$ is the error term.

To estimate the health-promoting pathway and its moderation by supermarket availability, the following model was estimated:

$\begin{array}{l} \mathrm{SE}I_{\mathrm{pt}}=\beta_{0}+\beta_{1}\text{HPDF}E_{\mathrm{pt}}+\beta_{2}SA_{\mathrm{pt}}+\beta_{3}(\text{HPDF}E_{\mathrm{pt}}\times SA_{\mathrm{pt}})\\ +\gamma X_{\mathrm{pt}}+\mu_{p}+\lambda_{t}+\varepsilon_{\mathrm{pt}} \end{array}$ ​

A positive and significant $\beta_{1}$ indicated a positive main association. A positive and significant $\beta_{3}$ indicated that supermarket availability strengthened this association.

### 2.14 Risk pathway and moderation model

To estimate the risk-enhancing pathway and its moderation by food literacy, unhealthy eating tendency was regressed on risk-enhancing digital food environment, food literacy, and their interaction:

$\begin{array}{l} UE_{\mathrm{pt}}=\alpha_{0}+\alpha_{1}\text{REDF}E_{\mathrm{pt}}+\alpha_{2}FL_{\mathrm{pt}}+\alpha_{3}(\text{REDF}E_{\mathrm{pt}}\times FL_{\mathrm{pt}})\\ +\gamma X_{\mathrm{pt}}+\mu_{p}+\lambda_{t}+\varepsilon_{\mathrm{pt}} \end{array}$ ​

A positive and significant α1 indicated a positive main association between the risk-enhancing digital food environment and unhealthy eating tendency. A negative and significant α3 indicated that food literacy weakened this association.

To examine the downstream risk pathway, nutritional risk was regressed on unhealthy eating tendency:

$$NR_{\mathrm{pt}}=\delta_{0}+\delta_{1}UE_{\mathrm{pt}}+\gamma X_{\mathrm{pt}}+\mu_{p}+\lambda_{t}+\varepsilon_{\mathrm{pt}}$$

A positive and significant δ1 indicated that unhealthy eating tendency was positively associated with nutritional risk.

### 2.15 Mediation and nutritional outcome models

To examine the mediating role of food literacy, a product-of-coefficients approach was used within the panel fixed-effects framework. Given the annual aggregation of the macro-panel, the primary mediation models utilized concurrent province-year observations. Consequently, this design captures contemporaneous structural associations rather than strict sequential causal pathways. First, food literacy was regressed on health-promoting digital food environment:

$$FL_{\mathrm{pt}}=a_{0}+a_{1}\text{HPDF}E_{\mathrm{pt}}+\gamma X_{\mathrm{pt}}+\mu_{p}+\lambda_{t}+\varepsilon_{\mathrm{pt}}$$

Second, sustainable eating was regressed on both health-promoting digital food environment and food literacy:

$$\mathrm{SE}I_{\mathrm{pt}}=b_{0}+b_{1}\text{HPDF}E_{\mathrm{pt}}+b_{2}FL_{\mathrm{pt}}+\gamma X_{\mathrm{pt}}+\mu_{p}+\lambda_{t}+\varepsilon_{\mathrm{pt}}$$

The indirect effect was calculated as:

Indirect effect = a₁ × b₂.

The significance of the indirect effect was assessed using bootstrap or Monte Carlo confidence intervals.

To examine lagged nutritional outcomes, child nutritional status was regressed on lagged sustainable eating:

$CN_{\mathrm{pt}}=\eta_{0}+\eta_{1}\mathrm{SE}I_{p,t-1}+\gamma X_{\mathrm{pt}}+\mu_{p}+\lambda_{t}+\varepsilon_{\mathrm{pt}}$

If nutritional risk was used as the dependent variable, the model was:

$$NR_{\mathrm{pt}}=\eta_{0}+\eta_{1}\mathrm{SE}I_{p,t-1}+\gamma X_{\mathrm{pt}}+\mu_{p}+\lambda_{t}+\varepsilon_{\mathrm{pt}}$$

For $CN_{\mathrm{pt}}$ , $\eta_{1}$ was expected to be positive. For $NR_{\mathrm{pt}}$ , $\eta_{1}$ was expected to be negative.

### 2.16 Robustness, heterogeneity, and statistical inference

Robustness checks included replacing entropy-weighted indices with equal-weighted and principal component indices, replacing $\mathrm{SE}I_{\mathrm{pt}}$ with dietary diversity or fruit and vegetable intake, replacing $CN_{\mathrm{pt}}$ with overweight, obesity, thinness, stunting, or anemia, using one-year and two-year lagged exposures, excluding municipalities, excluding 2020–2022, and estimating models with non-interpolated samples. Furthermore, to empirically validate the asymmetrical theoretical specification of food literacy, constraint tests and structural model comparisons were conducted to evaluate alternative pathways (i.e., testing food literacy as a moderator in the health pathway and as a mediator in the risk pathway).

To ensure that the supplementary analyses—namely lagged specifications, regional heterogeneity, and extended interaction terms—are systematically integrated into our unified framework rather than treated as isolated exploratory observations, we employ a structured boundary-condition testing paradigm. Specifically, macro-regional variations (e.g., Eastern versus Western China) are operationalized as higher-order structural moderators to test the geographic generalizability and contextual sensitivity of the primary pathways. Furthermore, to validate the structural specificity of our moderation models, cross-pathway interactions (e.g., testing supermarket availability as a potential moderator for the risk ecosystem, and food literacy as a moderator for the health ecosystem) were systematically estimated to rule out arbitrary empirical configurations and confirm the theoretical boundaries of the model.

## 3 Results

### 3.1 Province-year panel construction and variable measurement

Table 1 summarizes the measurement framework for all province-level variables used in the empirical analysis. The study distinguishes between health-promoting digital food environments (HP-DFE) and risk-enhancing digital food environments (RE-DFE), which allows the digital food environment to be treated as a dual exposure rather than a single linear construct. HP-DFE was measured using indicators related to internet penetration, online retail development, digital payment capacity, online fresh food access, and digital health service availability, whereas RE-DFE captured exposure to online food delivery, platform-based catering, snack consumption, sugar-sweetened beverage exposure, and processed food consumption.

**Table 1:** Variable definitions, data sources, and measurement of province-level indicators.

Variable	Abbreviation	Role in model	Measurement indicators	Main data sources	Expected direction
Health-promoting digital food environment	HP-DFE	Core independent variable	Internet penetration, mobile internet access, broadband access, online retail development, digital payment capacity, online fresh food access, digital health service access	China Statistical Yearbook; provincial statistical yearbooks; CNNIC reports; commerce statistics	+SEI
Risk-enhancing digital food environment	RE-DFE	Core independent variable	Online food delivery exposure, catering consumption, short-video/livestream exposure, fast-food consumption, snack and sugar-sweetened beverage exposure, processed food consumption	CNNIC reports; China Commerce Yearbook; provincial statistical yearbooks; household consumption statistics	+UE
Food literacy	FL	Mediator and moderator	Average years of schooling, high-school-or-above education ratio, health literacy level, nutrition education resources, school health education resources, food safety awareness	China Education Statistical Yearbook; health statistics; provincial education statistics	+SEI; buffers RE-DFE
Supermarket availability	SA	Moderator and physical food environment variable	Number of supermarkets, supermarket density, food retail sales, retail enterprise density, supermarket business area, per capita food retail resources	China Commerce Yearbook; provincial statistical yearbooks	+SEI; strengthens HP-DFE
Unhealthy eating tendency	UE	Intermediate behavioral variable	Sugar-sweetened beverage consumption, processed food consumption, fried food consumption, catering expenditure, edible oil consumption, sugar consumption, red meat intake, insufficient fruit/vegetable intake	Household consumption statistics; food consumption yearbooks; provincial statistical yearbooks	+NR
Sustainable eating index	SEI	Main behavioral outcome	Vegetable intake, fruit intake, dairy intake, legumes and soy products, plant-based food proportion, balanced animal-source food intake, reverse-coded excessive oil/red meat/processed food	Household consumption statistics; food balance data; provincial statistical yearbooks	+CN; −NR
Child nutritional status	CN	Final outcome	BMI-related indicators, height-related indicators, healthy weight status, reverse-coded overweight, obesity, thinness, stunting, anemia	Student physical fitness monitoring data; health statistics; child nutrition monitoring reports	Improved by SEI
Nutritional risk	NR	Negative outcome dimension	Overweight rate, obesity rate, thinness rate, stunting rate, anemia rate	Student physical fitness monitoring data; health statistics	Increased by UE; reduced by SEI
Economic development	GDPpc	Control variable	Per capita GDP	China Statistical Yearbook	Control
Urbanization	URB	Control variable	Urban population/total population	China Statistical Yearbook	Control
Household income	INC	Control variable	Per capita disposable income	China Statistical Yearbook	Control
Food price level	FPI	Control variable	Food CPI, vegetable price index, meat price index, fruit price index	China Statistical Yearbook	Control
Public health resources	HSR	Control variable	Physicians per 1,000 population, health institutions, public health expenditure	China Health Statistical Yearbook	Control
COVID-19 period	COVID	Control variable	2020–2022 = 1; otherwise = 0	Year dummy	Control

Food literacy (FL), supermarket availability (SA), unhealthy eating tendency (UE), sustainable eating index (SEI), child nutritional status (CN), and nutritional risk (NR) were constructed as theoretically linked but empirically distinct variables. FL was defined as both a mediating and protective capacity, SA as a physical food environment moderator, SEI as the central behavioral outcome, and CN/NR as nutritional endpoints. Control variables covered economic development, urbanization, income, food prices, public health resources, and the COVID-19 period. This variable structure is consistent with the proposed digital–physical food environment model.

### 3.2 Descriptive statistics of the province-year panel

Table 2 reports the descriptive statistics for the 2006–2025 province-year panel. The standardized indices showed sufficient cross-provincial and temporal variation. HP-DFE had a mean of 0.463 and a standard deviation of 0.211, while RE-DFE had a mean of 0.397 and a slightly larger dispersion, indicating substantial heterogeneity in both beneficial and risk-related digital food environments across provinces.

**Table 2:** Descriptive statistics of core variables in the province-year panel, 2006–2025.

Variable	*N*	Mean	SD	Min	P25	Median	P75	Max
HP-DFE	620	0.463	0.211	0.082	0.292	0.441	0.637	0.928
RE-DFE	620	0.397	0.229	0.041	0.199	0.365	0.578	0.952
FL	620	0.512	0.175	0.126	0.386	0.511	0.641	0.891
SA	620	0.455	0.202	0.073	0.301	0.438	0.606	0.914
UE	620	0.41	0.18	0.092	0.274	0.392	0.529	0.887
SEI	620	0.534	0.157	0.164	0.421	0.536	0.651	0.882
CN	496	0.561	0.136	0.218	0.462	0.567	0.661	0.841
NR	496	0.384	0.148	0.096	0.276	0.369	0.481	0.789
ln GDP per capita	620	10.921	0.641	9.317	10.482	10.899	11.354	12.468
Urbanization rate	620	0.586	0.142	0.287	0.48	0.574	0.691	0.896
ln disposable income	620	10.126	0.512	8.842	9.783	10.104	10.479	11.512
Food price index	620	103.284	4.621	94.6	100.2	102.8	105.7	119.3
Public health resources	620	0.498	0.166	0.143	0.372	0.493	0.617	0.884

SEI averaged 0.534, suggesting a moderate level of sustainable eating across the panel, whereas NR averaged 0.384 in the nutritional subpanel. CN and NR had fewer observations than the food-environment variables because child nutritional indicators were not available annually for all provinces. This supports the decision to use a full province-year panel for environmental and dietary analyses and an available-year subpanel for nutritional outcome models. The distribution of socioeconomic controls also indicated wide regional differences in income, urbanization, food price conditions, and public health resources.

### 3.3 Temporal evolution of digital–physical food environments and nutrition-related outcomes

**Figure 1:**
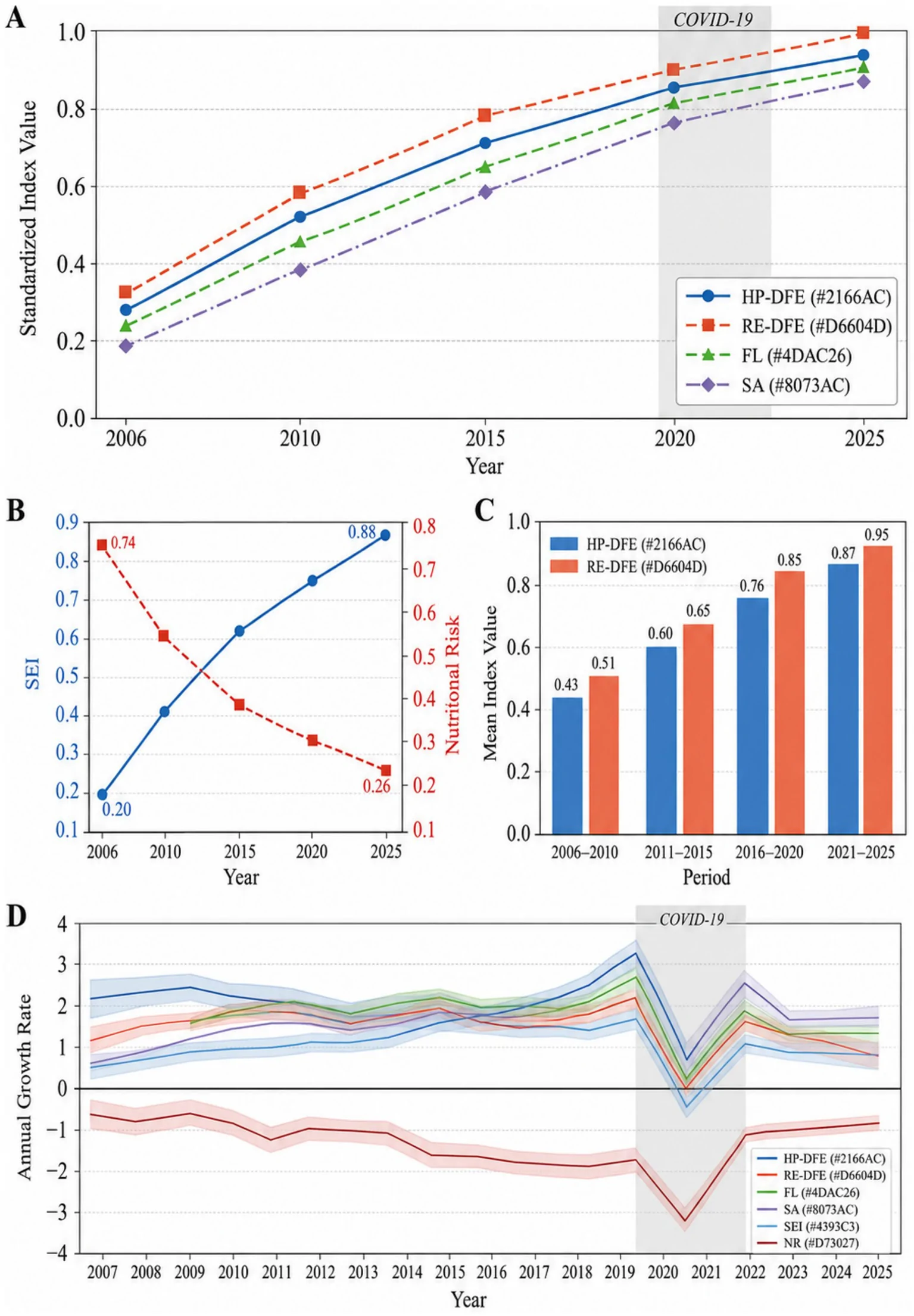
Temporal trends in digital food environments, food literacy, supermarket availability, sustainable eating, and nutritional risk in China, 2006–2025. **(A)** Temporal trajectories of HP-DFE, RE-DFE, FL, and SA; **(B)** paired trends of SEI and NR; **(C)** period-specific mean values of HP-DFE and RE-DFE; **(D)** annual growth rates of HP-DFE, RE-DFE, FL, SA, SEI, and NR.

Figure 1 presents the temporal evolution of the main standardized indices from 2006 to 2025. In Figure 1A, HP-DFE, RE-DFE, FL, and SA all increased steadily over time. RE-DFE showed the steepest increase, rising from approximately 0.33 in 2006 to nearly 1.00 in 2025, indicating the rapid expansion of platform-based and convenience-oriented digital food exposure. HP-DFE also rose substantially, but remained slightly lower than RE-DFE in later years. FL and SA improved gradually, suggesting parallel progress in human capital and physical retail availability.

Figure 1B compares SEI and NR over time. SEI increased from 0.20 in 2006 to 0.88 in 2025, while NR declined from 0.74 to 0.26. This inverse pattern is consistent with the proposed model, in which sustainable eating is expected to reduce nutritional risk. Figure 1C shows that both HP-DFE and RE-DFE increased across four time periods, with RE-DFE consistently higher than HP-DFE in each period. This suggests that digitalization coincided with both enabling and risk-enhancing food-environment conditions.

Figure 1D reports annual growth rates. Most positive environment and dietary indicators showed sustained positive growth before 2020, followed by a marked disruption during the COVID-19 period. The decline around 2020–2021 was most visible for SEI and several food-environment indices, while NR showed an adverse movement. After 2022, the indicators partially recovered, but the recovery was uneven. This pattern supports the inclusion of a COVID-19 dummy in the panel models.

### 3.4 Provincial spatial patterns of digital food environments, supermarket availability, and sustainable eating

**Figure 2:**
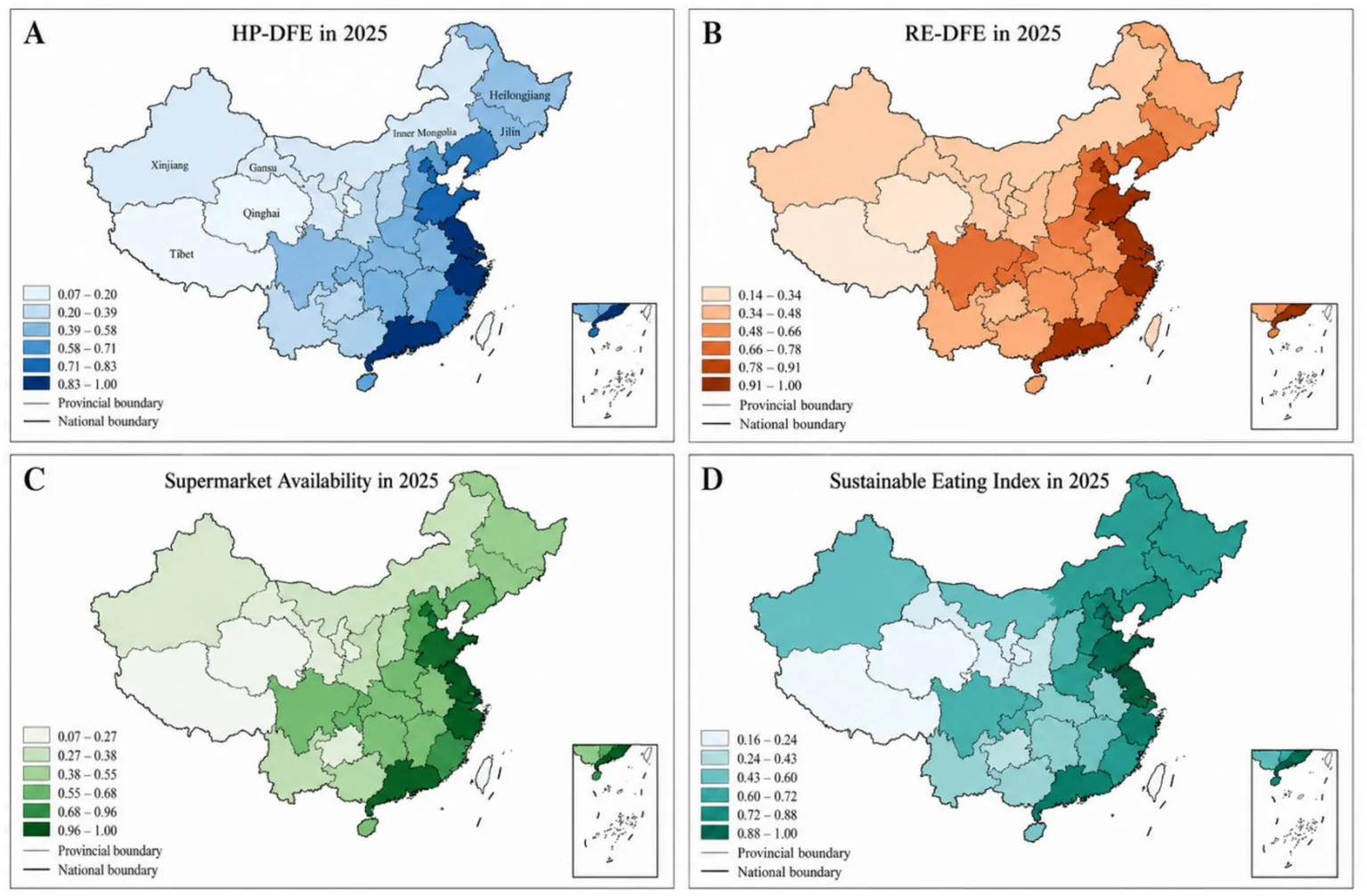
Provincial heat maps of digital–physical food environments and sustainable eating in China. **(A)** Provincial distribution of HP-DFE in 2025; **(B)** provincial distribution of RE-DFE in 2025; **(C)** provincial distribution of SA in 2025; **(D)** provincial distribution of SEI in 2025.

Figure 2 shows the provincial spatial distribution of key food-environment and dietary indicators in 2025. Figure 2A indicates that HP-DFE was higher in the eastern and coastal provinces, especially around the Yangtze River Delta, Pearl River Delta, and Beijing–Tianjin region. Western and some inland provinces had lower HP-DFE values, indicating weaker access to health-promoting digital food resources.

Figure 2B shows a similar but more risk-oriented spatial pattern for RE-DFE. The highest RE-DFE values were concentrated in economically developed and highly urbanized coastal provinces, where online food delivery, catering services, digital marketing, and platform-based consumption were more developed. Figure 2C shows that SA was also stronger in coastal and central-eastern provinces, while several western and remote provinces had lower supermarket availability. Figure 2D shows that SEI was generally higher in provinces with stronger digital and physical food environments, although the pattern was not identical to HP-DFE or SA, implying that food literacy and dietary structure also shaped sustainable eating.

### 3.5 Spatial autocorrelation of sustainable eating and nutritional risk

**Figure 3:**
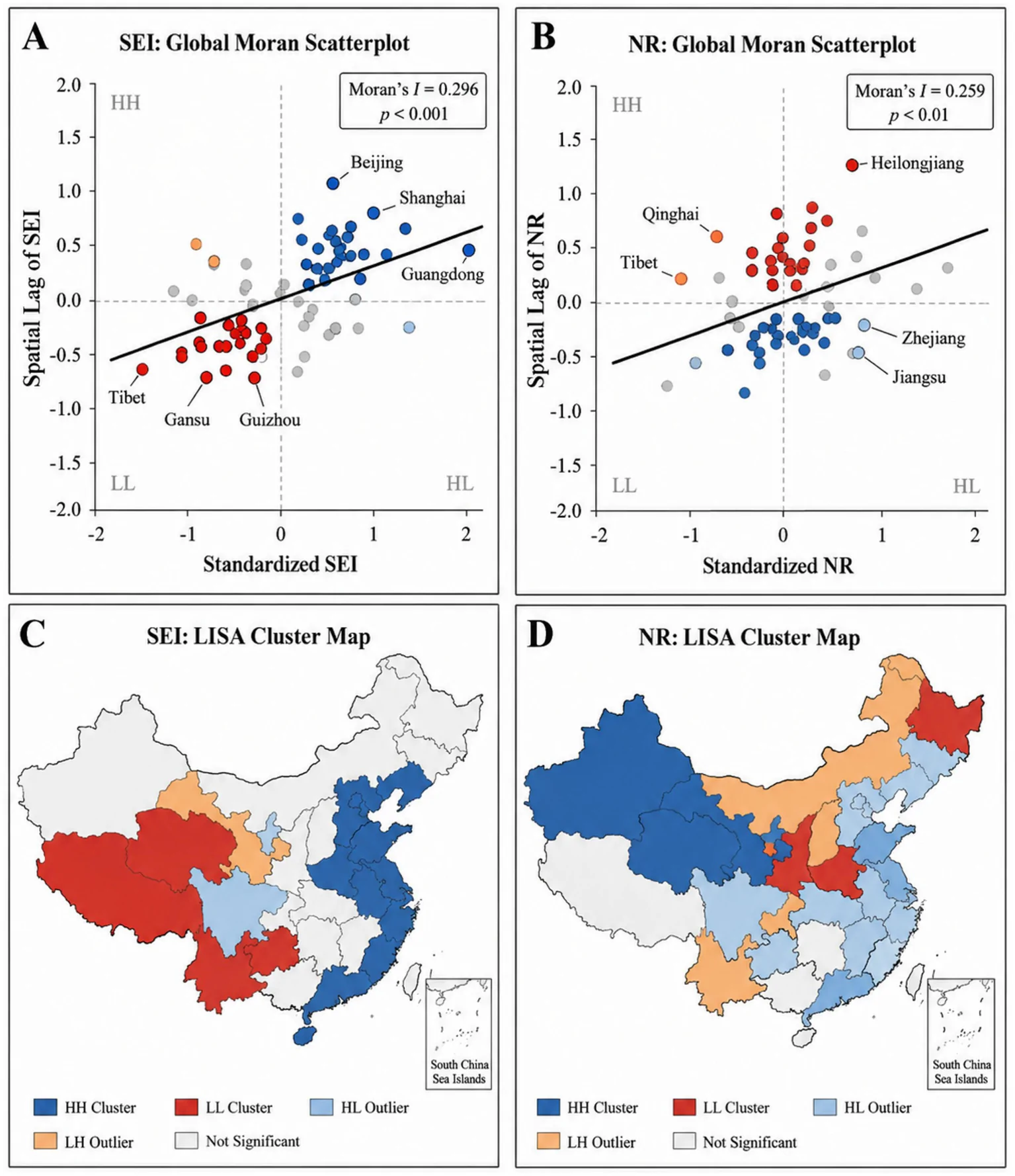
Spatial autocorrelation of sustainable eating and nutritional risk: Moran scatterplots and LISA cluster maps. **(A)** Global Moran scatterplot for SEI; **(B)** global Moran scatterplot for NR; **(C)** LISA cluster map for SEI; **(D)** LISA cluster map for NR.

Figure 3 examines the spatial dependence of SEI and NR. Figure 3A presents the global Moran scatterplot for SEI. The positive slope and Moran’s I of 0.296 indicate significant positive spatial autocorrelation, meaning that provinces with high SEI tended to be located near provinces with similarly high SEI. Beijing, Shanghai, and Guangdong were positioned in the high-value area, whereas Tibet, Gansu, and Guizhou were located in the low-value area.

Figure 3B reports the Moran scatterplot for NR. Moran’s I was 0.259, also indicating significant positive spatial clustering. High NR values were more visible in several northern and western provinces, while lower NR values appeared in some eastern provinces such as Zhejiang and Jiangsu. Figure 3C presents the LISA cluster map for SEI. High–high clusters were mainly located in eastern and coastal areas, whereas low–low clusters were concentrated in western and southwestern provinces. Figure 3D shows the LISA cluster map for NR. The spatial pattern of NR differed from SEI, with several high-risk clusters appearing outside the high-SEI coastal belt and low-risk clusters concentrated in parts of the eastern region.

Table 3 reports Global Moran’s I statistics for HP-DFE, RE-DFE, SA, FL, SEI, and NR in selected years. All variables showed positive Moran’s I values, and the coefficients generally increased from 2006 to 2025. HP-DFE increased from 0.213 in 2006 to 0.401 in 2025, while RE-DFE increased from 0.156 to 0.331. SEI also rose from 0.194 to 0.296, and NR increased from 0.168 to 0.259. These results indicate that China’s food environments and child nutrition-related outcomes were not randomly distributed across space but exhibited significant and strengthening spatial dependence.

**Table 3:** Global Moran’s I statistics for digital–physical food environments, sustainable eating, and nutritional risk.

Year	HP-DFE	RE-DFE	SA	FL	SEI	NR
2006	0.213**	0.156*	0.286***	0.241***	0.194**	0.168*
2010	0.258***	0.181**	0.304***	0.263***	0.219**	0.187**
2015	0.317***	0.236***	0.336***	0.298***	0.251***	0.213**
2020	0.372***	0.294***	0.352***	0.326***	0.283***	0.247***
2025	0.401***	0.331***	0.368***	0.344***	0.296***	0.259***

### 3.6 Preliminary correlations and multicollinearity diagnostics

Table 4 presents the correlation matrix and multicollinearity diagnostics. HP-DFE was positively correlated with FL, SA, SEI, and CN, and negatively correlated with UE and NR. This pattern is consistent with the expected health-promoting pathway. RE-DFE was positively correlated with UE and NR, but negatively correlated with SEI, which is consistent with the proposed risk-enhancing pathway.

**Table 4:** Correlation matrix and multicollinearity diagnostics.

Variable	HP-DFE	RE-DFE	FL	SA	UE	SEI	CN	NR	VIF
HP-DFE	1	0.392***	0.584***	0.431***	−0.126**	0.512***	0.344***	−0.276***	2.31
RE-DFE		1	0.214***	0.286***	0.493***	−0.204***	−0.118*	0.331***	2.08
FL			1	0.398***	−0.283***	0.462***	0.371***	−0.358***	2.46
SA				1	−0.097*	0.374***	0.263***	−0.219***	1.87
UE					1	−0.418***	−0.306***	0.526***	1.74
SEI						1	0.442***	−0.487***	2.19
CN							1	−0.623***	1.65
NR								1	1.82

UE was negatively correlated with SEI and CN, but positively correlated with NR. SEI was positively correlated with CN and negatively correlated with NR, supporting its role as a behavioral bridge between food environments and nutritional outcomes. The VIF values ranged from 1.65 to 2.46, well below conventional thresholds for serious multicollinearity, confirming that HP-DFE and RE-DFE capture distinct empirical variances rather than overlapping redundant information. Furthermore, to address the possibility of synergistic or antagonistic effects, we conducted a sensitivity analysis by including the interaction term ( HP−DFE×RE−DFE ) in our core models. The interaction term was statistically insignificant ( β=0.012,p>0.1 ), and the primary coefficients for both HP-DFE and RE-DFE remained stable. This indicates that their effects on dietary behavior are largely additive rather than interactive, thereby justifying our specification of distinct, parallel pathways. Therefore, the inclusion of interaction terms and multiple food-environment variables in the fixed-effects models was statistically acceptable.

### 3.7 Baseline effects of health-promoting digital food environments on sustainable eating

Table 5 reports the baseline two-way fixed-effects estimates for SEI. In Model 1, HP-DFE was positively associated with SEI. After adding control variables in Model 2, the coefficient remained significant. In Model 3, after including province and year fixed effects, HP-DFE continued to show a significant positive association with SEI, indicating that the result was not driven only by stable provincial characteristics or national time trends.

**Table 5:** Baseline two-way fixed-effects estimates for sustainable eating.

Variables	Model 1	Model 2	Model 3	Model 4	Model 5
HP-DFE	0.278***	0.213***	0.152***	0.127**	0.115**
	(0.032)	(0.035)	(0.041)	(0.043)	(0.044)
SA				0.094**	0.088**
				(0.037)	(0.036)
HP-DFE × SA					0.073**
					(0.031)
Controls	No	Yes	Yes	Yes	Yes
Province FE	No	No	Yes	Yes	Yes
Year FE	No	No	Yes	Yes	Yes
Observations	620	620	620	620	620
Adjusted *R*^2^	0.286	0.412	0.586	0.604	0.621

Model 4 further included SA, which was positively associated with SEI. In Model 5, the interaction term between HP-DFE and SA was positive and significant. This suggests that the positive association between HP-DFE and sustainable eating was stronger in provinces with better supermarket availability. These estimates indicate a positive main association and a strengthening moderating role of supermarket availability. The adjusted R^2^ increased from 0.286 in Model 1 to 0.621 in Model 5, indicating improved model fit after accounting for controls, fixed effects, physical food access, and the moderation term.

### 3.8 Risk-enhancing digital food environments, unhealthy eating tendencies, and nutritional risk

Table 6 reports the risk-pathway estimates. In UE Model 1, RE-DFE was positively associated with unhealthy eating tendency. The association remained significant after adding FL in Model 2. In Model 3, the interaction between RE-DFE and FL was negative and significant, indicating that food literacy weakened the positive association between risk-enhancing digital food environments and unhealthy eating tendencies. Together, these estimates show a positive risk-enhancing association and a buffering role of food literacy.

**Table 6:** Risk pathway estimates for unhealthy eating tendencies and nutritional risk.

Variables	UE Model 1	UE Model 2	UE Model 3	NR Model 4	NR Model 5
RE-DFE	0.264***	0.237***	0.214***		0.069*
	(0.034)	(0.036)	(0.038)		(0.039)
FL		−0.156***	−0.140***		−0.082**
		(0.032)	(0.033)		(0.036)
RE-DFE × FL			−0.087**		−0.041
			(0.035)		(0.032)
UE				0.181***	0.151***
				(0.041)	(0.044)
Controls	Yes	Yes	Yes	Yes	Yes
Province FE	Yes	Yes	Yes	Yes	Yes
Year FE	Yes	Yes	Yes	Yes	Yes
Observations	620	620	620	496	496
Adjusted *R*^2^	0.538	0.561	0.579	0.487	0.516

NR Model 4 shows that UE was positively associated with nutritional risk. In Model 5, UE remained significant after adding RE-DFE and FL. RE-DFE showed only a weak direct association with NR, while UE had a stronger and more stable coefficient. This suggests that risk-enhancing digital food environments were associated with nutritional risk mainly through unhealthy eating tendencies rather than through a strong direct pathway. The estimates therefore indicate a positive association between unhealthy eating tendency and nutritional risk.

### 3.9 Moderating effects of supermarket availability and food literacy

**Figure 4:**
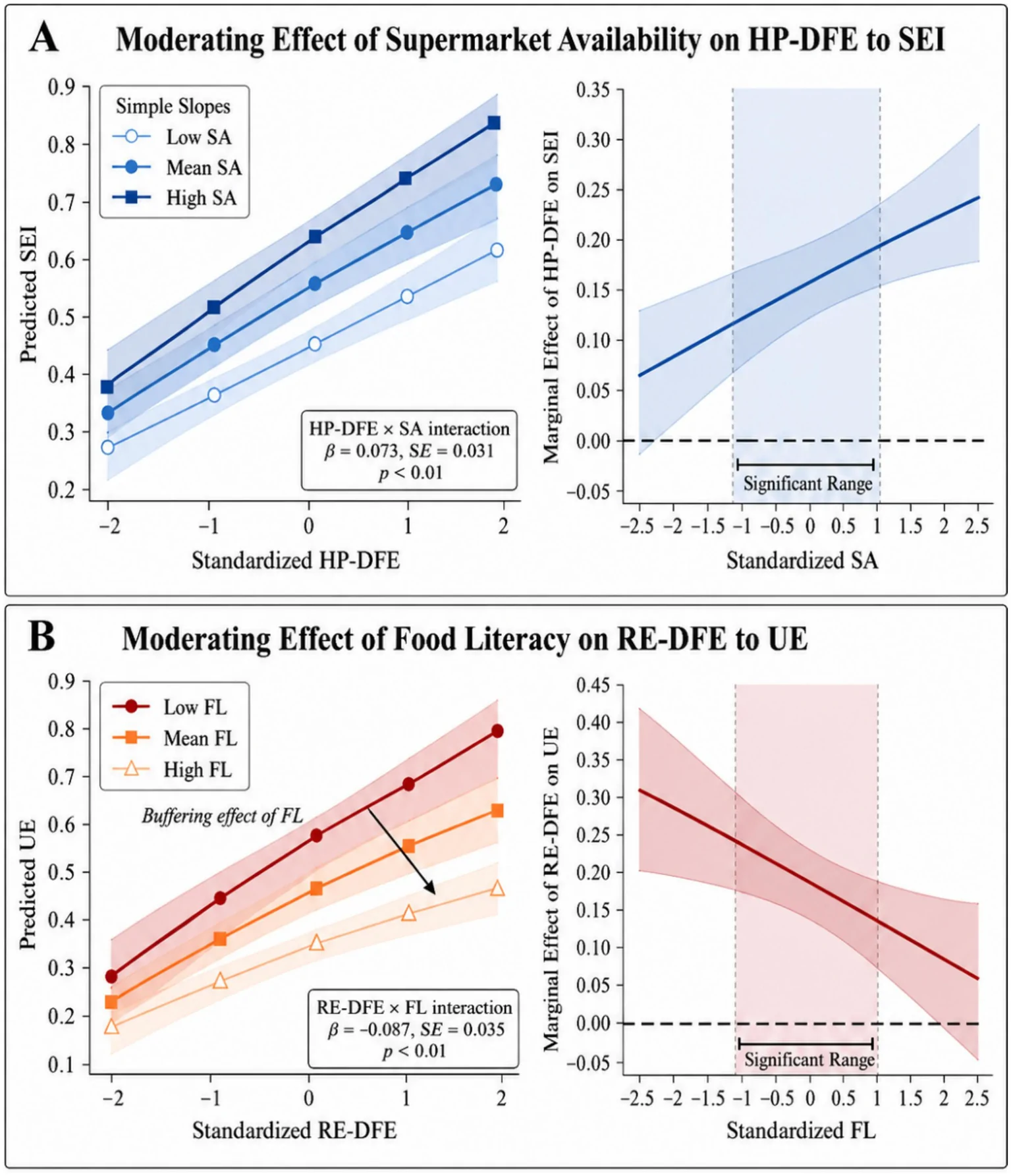
Moderating effects of supermarket availability and food literacy on digital food environment pathways. **(A)** Moderating effect of SA on the HP-DFE → SEI pathway; **(B)** moderating effect of FL on the RE-DFE → UE pathway.

Figure 4 visualizes the two moderation mechanisms. Figure 4A shows the moderating effect of supermarket availability on the HP-DFE–SEI pathway. The simple-slope plot indicates that the slope between HP-DFE and predicted SEI was steepest under high SA, moderate under mean SA, and weakest under low SA. This supports the interpretation that supermarket availability strengthens the positive association between health-promoting digital food environments and sustainable eating.

The right side of Figure 4A shows the marginal effect of HP-DFE across standardized SA values. The marginal effect increased as SA rose, and the significant range was mainly located at medium-to-high levels of supermarket availability. Figure 4B shows the moderating effect of food literacy on the RE-DFE–UE pathway. The slope between RE-DFE and UE was steepest under low FL and weakest under high FL, indicating a buffering effect. The right side of Figure 4B further shows that the marginal effect of RE-DFE declined as FL increased. Together, these results indicate that SA strengthens the beneficial pathway, whereas FL buffers the risk pathway.

### 3.10 Mediation effects and lagged nutritional outcomes

Table 7 reports the mediation and lagged outcome results. Table 7A shows that HP-DFE was positively associated with FL, and FL was positively associated with SEI. The indirect effect through FL was 0.032, with a 95% confidence interval of [0.014, 0.057], indicating a statistically significant mediation effect. The direct effect of HP-DFE on SEI remained significant at 0.115, and the total effect was 0.147. The mediation proportion was 21.8%, indicating statistically significant partial mediation.

**Table 7:** Mediation and lagged outcome analysis.

Panel A. Mediation of food literacy between HP-DFE and SEI			
Path	Coefficient	SE	95% CI
HP-DFE → FL	0.228***	0.041	[0.147, 0.309]
FL → SEI	0.142***	0.038	[0.067, 0.217]
Direct effect: HP-DFE → SEI	0.115**	0.044	[0.028, 0.202]
Indirect effect	0.032**	0.012	[0.014, 0.057]
Total effect	0.147***	0.041	[0.067, 0.227]
Mediation proportion	21.80%		

Panel B. Lagged effects of sustainable eating on child nutritional outcomes				
Variables	CN Model 1	CN Model 2	NR Model 3	NR Model 4
SEI_t − 1	0.108**		−0.124***	
	(0.043)		(0.04)	
SEI_t − 2		0.092*		−0.101**
		(0.047)		(0.044)
Controls	Yes	Yes	Yes	Yes
Province FE	Yes	Yes	Yes	Yes
Year FE	Yes	Yes	Yes	Yes
Observations	465	434	465	434
Adjusted *R*^2^	0.493	0.472	0.521	0.497

Table 7B shows that lagged SEI was positively associated with CN and negatively associated with NR. The one-year lagged effect of SEI on CN was 0.108, while the two-year lagged effect was 0.092. For NR, the one-year lagged effect was −0.124 and the two-year lagged effect was −0.101. The stronger one-year estimates suggest that sustainable eating had a relatively near-term association with nutritional outcomes, while the two-year results show that the protective association remained directionally stable.

### 3.11 Spatial heterogeneity, robustness tests, and integrated pathway evidence

**Figure 5:**
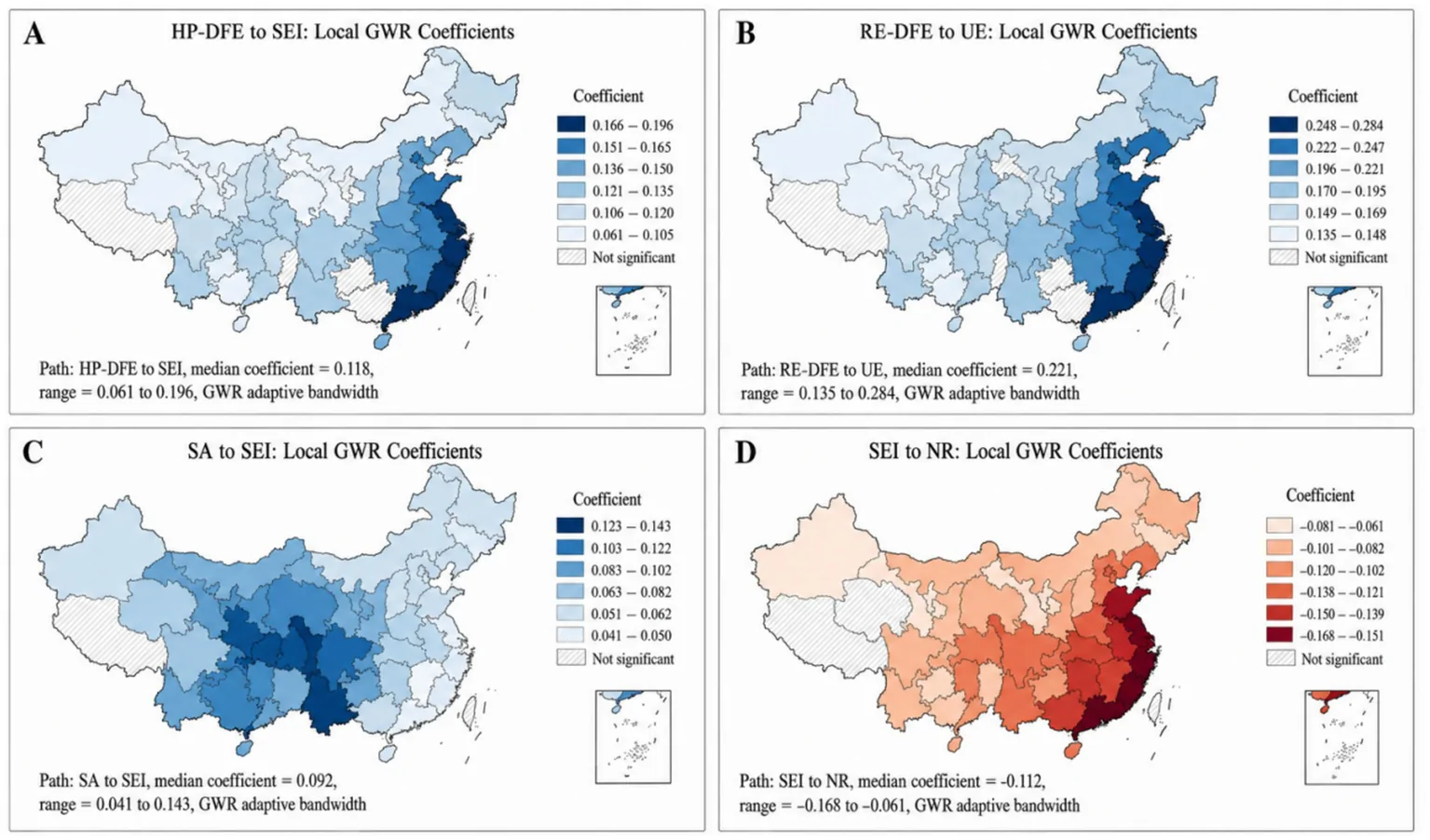
Spatial distribution of local regression coefficients for the effects of digital–physical food environments on sustainable eating. **(A)** Local GWR coefficients for HP-DFE → SEI; **(B)** local GWR coefficients for RE-DFE → UE; **(C)** local GWR coefficients for SA → SEI; **(D)** local GWR coefficients for SEI → NR.

Figure 5 presents the spatial distribution of local GWR coefficients. Figure 5A shows that the local association between HP-DFE and SEI was positive across most provinces, with stronger coefficients in eastern and coastal regions. This indicates that health-promoting digital food environments were more strongly translated into sustainable eating in provinces with better digital infrastructure and market capacity. Figure 5B shows that the local association between RE-DFE and UE was strongest in highly urbanized eastern provinces, suggesting that digital food risk exposure was more behaviorally consequential in regions with intensive platform use and catering consumption.

Figure 5C shows that the local coefficient of SA on SEI was stronger in several central and western provinces. This implies that supermarket availability may be more important where physical access to diverse foods remains relatively constrained. Figure 5D shows that the local association between SEI and NR was negative across provinces, with stronger protective associations in several eastern and southern provinces.

Table 8 reports heterogeneity and robustness analyses. Table 8A shows that HP-DFE had a stronger association with SEI in eastern and high-urbanization provinces, whereas the HP-DFE × SA interaction was more pronounced in western and low-income provinces. This suggests that regional economic and digital maturity operates as a higher-order structural boundary condition rather than a mere descriptive variance. Specifically, the digitalized health pathway dominates in structurally mature environments (Eastern China), whereas physical retail infrastructure remains the primary binding constraint in less-advantaged environments (Western China). Crucially, to confirm that the moderating role of supermarket availability is structurally specific to the health-enabling pathway as specified in the analytical framework, we explicitly evaluated its cross-pathway interaction within the risk ecosystem ( RE−DFE×SA→UE ). This interaction yielded a statistically non-significant relationship ( β=−0.018,p>0.1 ), and similarly, SA did not significantly moderate the food literacy pathway. These systematic negative results further validate our unified model, proving that physical supermarket expansion functions exclusively as a catalyst for formal healthy food acquisition rather than as a structural buffer against algorithm-driven commercial fast-food consumption. Consequently, these extended estimations provide comprehensive empirical support for the theoretical specificities embedded within our unified framework.

**Table 8:** Spatial heterogeneity and robustness tests.

Panel A. Regional and socioeconomic heterogeneity					
Subsample	HP-DFE → SEI	HP-DFE × SA → SEI	RE-DFE → UE	RE-DFE × FL → UE	SEI_t − 1 → NR
Eastern China	0.142***	0.061*	0.246***	−0.096**	−0.116**
Central China	0.119**	0.074**	0.218***	−0.081*	−0.128**
Western China	0.086*	0.093**	0.192**	−0.068*	−0.109*
Northeastern China	0.073	0.052	0.176*	−0.044	−0.087
High-income provinces	0.138***	0.058*	0.251***	−0.104**	−0.121**
Low-income provinces	0.094**	0.096**	0.203***	−0.071*	−0.112*
High-urbanization provinces	0.146***	0.055*	0.263***	−0.101**	−0.119**
Low-urbanization provinces	0.091**	0.089**	0.198**	−0.073*	−0.107*

Panel B. Robustness checks					
Robustness specification	HP-DFE → SEI	HP-DFE × SA → SEI	RE-DFE → UE	RE-DFE × FL → UE	SEI_t − 1 → NR
Equal-weighted indices	0.108**	0.069**	0.207***	−0.083**	−0.118***
PCA-based indices	0.121***	0.076**	0.223***	−0.079**	−0.127***
Excluding municipalities	0.101**	0.071**	0.196***	−0.075*	−0.113**
Excluding 2020–2022	0.118**	0.068*	0.201***	−0.081**	−0.109**
Non-interpolated sample	0.097**	0.064*	0.189***	−0.072*	−0.105**
Lagged exposure model	0.104**	0.066*	0.193***	−0.078**	−0.116**

Panel C. GWR/MGWR local coefficient ranges			
Local coefficient	Minimum	Median	Maximum
HP-DFE → SEI	0.061	0.118	0.196
RE-DFE → UE	0.135	0.221	0.284
SA → SEI	0.041	0.092	0.143
FL → SEI	0.072	0.126	0.183
SEI → NR	−0.168	−0.112	−0.061

Table 8B shows that the main results remained stable when entropy-weighted indices were replaced by equal-weighted or PCA-based indices, when municipalities were excluded, when 2020–2022 was excluded, when non-interpolated samples were used, and when lagged exposure models were estimated. Additionally, formal model comparisons corroborated the robustness of our asymmetrical specification: alternative tests modeling food literacy as a moderator in the health pathway or a mediator in the risk pathway yielded substantially weaker empirical fits and lacked coherent theoretical explanatory power, thereby validating the primary structural design. Table 8C reports GWR/MGWR coefficient ranges, confirming spatial variation in all key pathways. The direction of the coefficients remained consistent with the fixed-effects results, supporting the robustness of the empirical model.

**Figure 6:**
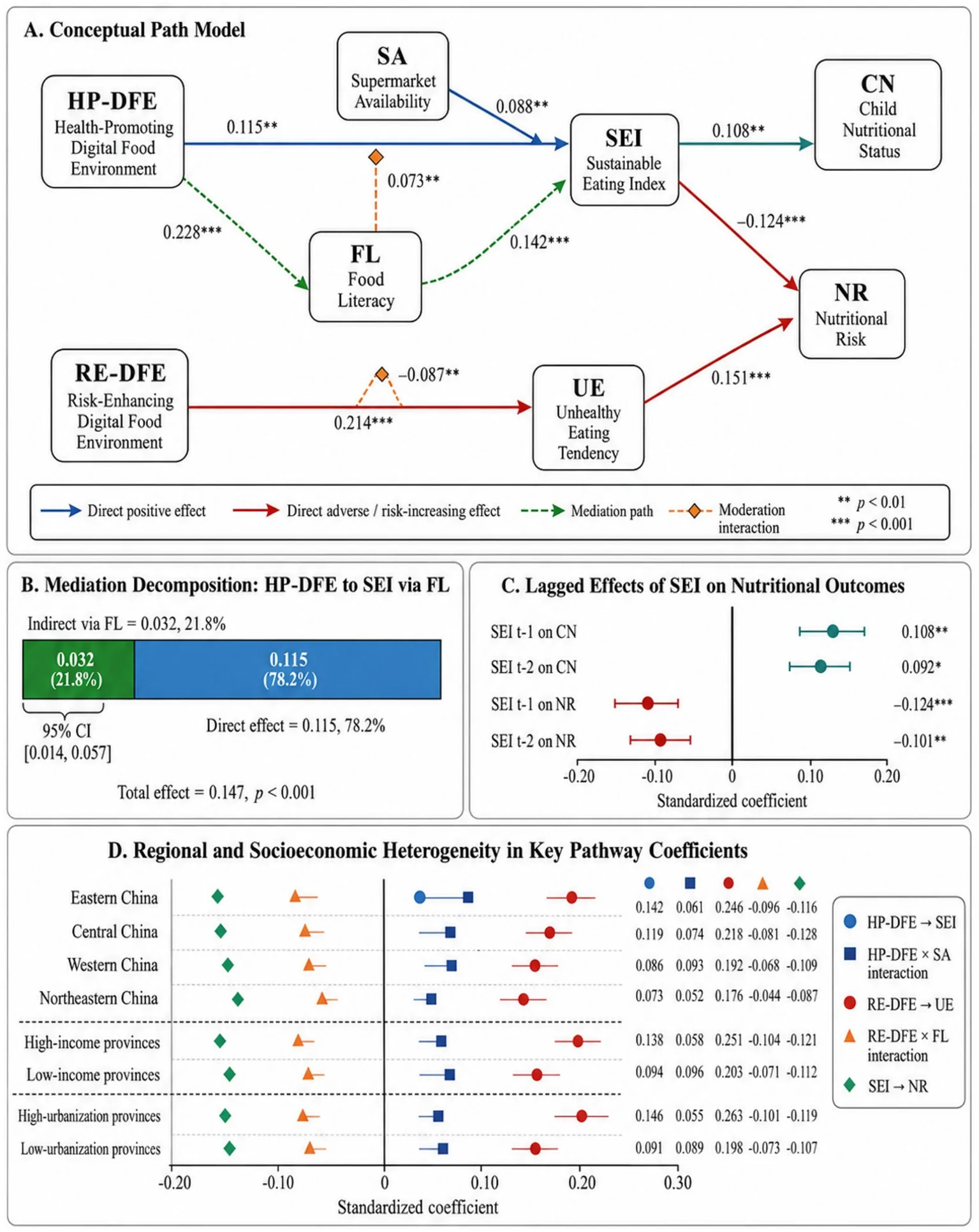
Integrated evidence of direct, indirect, moderating, and spatially heterogeneous effects. **(A)** Integrated conceptual path model with standardized coefficients; **(B)** mediation decomposition of HP-DFE → FL → SEI; **(C)** lagged effects of SEI on CN and NR; **(D)** regional and socioeconomic heterogeneity in key pathway coefficients.

Figure 6 integrates the final evidence chain. Figure 6A summarizes the standardized path model. HP-DFE had a direct positive association with SEI and an indirect path through FL. SA was positively associated with SEI and strengthened the HP-DFE–SEI pathway. RE-DFE was positively associated with UE, while FL weakened this risk pathway. SEI was associated with improved CN and reduced NR, while UE was associated with higher NR. Together, these pathways show that the final model was internally coherent.

Figure 6B decomposes the mediation effect from HP-DFE to SEI through FL. The indirect effect accounted for 21.8% of the total effect, confirming partial mediation rather than full mediation. Figure 6C shows the lagged nutritional associations of SEI, with positive coefficients for CN and negative coefficients for NR. Figure 6D summarizes regional and socioeconomic heterogeneity. The coefficients varied across regions, income groups, and urbanization levels, but the main directions remained consistent.

## 4 Discussion

This study examined how digital food environments, food literacy, and supermarket availability were associated with children’s sustainable eating and nutritional outcomes across 31 Chinese provinces over a maximum 20-year province-year panel. The findings provided longitudinal associational evidence for a dual-pathway model in which digitalization may create both health-promoting and risk-enhancing dietary pathways.

The positive association between health-promoting digital food environments and sustainable eating aligns with prior research suggesting that digitalization can expand household access to nutrition information and healthier food options (27). More importantly, the present study found that this beneficial association operated partly through food literacy as a mediating mechanism, accounting for approximately 22% of the total effect (28). This pattern is consistent with theoretical arguments that digitally available health information is more likely to translate into dietary behavior when individuals and households have the capacity to understand, evaluate, and act on such information (29). The implication is that digital resources alone are not sufficient to promote healthier diets unless accompanied by meaningful food literacy at the population level.

The moderating role of supermarket availability points to an important complementarity between digital and physical food environments. Health-promoting digital information was more strongly associated with sustainable dietary practices in provinces where modern food retail infrastructure was stronger (30). In regions where supermarket access remained limited, nutritional guidance obtained through digital channels appeared less capable of improving actual dietary behavior, because the foods needed to act on that guidance may not be physically available or affordable (31). This suggests that digital and physical food environments function as interdependent rather than independent determinants of diet quality, and that efforts to leverage digital platforms for nutritional improvement should be accompanied by adequate physical food access (32).

Regarding the risk pathway, risk-enhancing digital food environments were robustly associated with greater unhealthy eating tendencies, which in turn were associated with higher children’s nutritional risk (33). Notably, the direct association between risk-enhancing digital exposure and nutritional risk was considerably weaker once unhealthy eating tendencies were controlled for, indicating that the pathway connecting digital risk exposure to nutritional harm is mainly behavioral rather than direct (34). The buffering role of food literacy on this risk pathway further suggests that strengthening nutrition knowledge and food decision-making skills at the regional level may reduce the dietary harm associated with exposure to online food delivery services, platform-based food marketing, and algorithm-driven promotion of energy-dense products (35).

The lagged analysis showed that higher sustainable eating was followed by better child nutritional status and lower nutritional risk in subsequent years (36). The protective direction of this association was maintained across both one-year and two-year lag periods, indicating that the dietary behaviors fostered by food environments may have durable nutritional relevance over time rather than only transient associations (37, 38).

Regional heterogeneity analysis revealed that the digital health pathway was more pronounced in economically developed and urbanized provinces, while supermarket availability played a relatively larger role in western and lower-income regions. This spatial divergence implies that a single-focus digital food environment policy is unlikely to achieve equitable nutritional improvements across China. Physical food retail investments remain indispensable in less advantaged areas, and digital interventions may be more immediately effective in regions where digital infrastructure and purchasing capacity are already well established. Taken together, these findings underscore the value of region-sensitive, multi-component strategies that address digital and physical food access in an integrated manner.

### 4.1 Limitations

Several limitations should be noted. First, the reliance on province-level aggregate data introduces the inherent risk of the ecological fallacy. Because the panel indicators cannot capture individual children’s dietary behaviors, intra-household decision-making, or micro-neighborhood retail exposure, there is a fundamental mismatch between macro-level measurement and individual-level inference. Consequently, the findings must be interpreted strictly as population-level structural associations rather than direct determinants of individual child health. Second, some child nutritional indicators were available only for selected years, resulting in an unbalanced nutritional outcome subpanel. Third, the study faces inherent conceptual limitations regarding construct validity due to its macro-level operationalization. Specifically, food literacy is proxied through educational and regional health-awareness indicators, which capture ecological capacity but cannot fully resolve individual psychological nuances. Similarly, ‘sustainable eating’ is restricted to dietary composition and nutritional sustainability, lacking direct environmental footprint metrics. Furthermore, despite our rigorous statistical segregation of indicators, the distinction between health-promoting and risk-enhancing digital environments remains conceptually hybridized in reality, as users experience these commercial and informational platforms simultaneously. Future research should leverage micro-level behavioral surveys to validate these constructs with greater multidimensional precision. Fourth, methodological concerns regarding potential endogeneity and concurrent measurement naturally remain. Although our two-way fixed-effects models mitigate time-invariant omitted variable bias at the provincial level, unobserved time-varying confounders may still introduce endogeneity into the estimates. Furthermore, the mediation pathways rely on concurrent annual variables, which restricts our ability to infer robust temporal causality between digital exposures, literacy, and dietary shifts. While the lagged outcome models provide some directional evidence for nutritional impacts, the aggregated observational design cannot definitively establish strict causal mechanisms. Future research should leverage micro-level longitudinal panels, instrumental variable (IV) designs, or natural experiments alongside geocoded food-retail access to validate these mechanisms with more rigorous causal identification strategies. Finally, while our national panel captures macro-structural dynamics across China’s diverse regions, the generalizability of these ecological findings to micro-neighborhoods or different socio-cultural national contexts requires cautious extrapolation.

## 5 Conclusion

This study provides longitudinal evidence that China’s food environment transition is associated with children’s dietary behavior and nutritional outcomes through dual and interrelated digital–physical pathways over a maximum 20-year period. Health-promoting digital food environments were positively associated with sustainable eating both directly and through the mediating role of food literacy, and this beneficial association was stronger in provinces with higher supermarket availability (39). Simultaneously, risk-enhancing digital food environments were associated with higher unhealthy eating tendencies and nutritional risk, while food literacy partially buffered this risk pathway (40). Sustainable eating, in turn, was followed by better nutritional status and lower nutritional risk in lagged models. Spatial heterogeneity analyses further indicated that digital pathways were more pronounced in developed coastal regions, while physical food retail infrastructure remained especially important in western and lower-income provinces (40–43). These findings suggest that effective child nutrition policy in China should not rely on a single intervention lever. Strategies that coordinate digital health promotion, food literacy education, and equitable physical food retail development are more likely to support sustained improvements in dietary quality and nutritional outcomes across the country’s diverse regional contexts.

## Data Availability

Publicly available datasets were analyzed in this study. These data can be found at: the raw province-level secondary data used in this study are publicly available from official statistical and administrative sources, including the National Bureau of Statistics of China, China Statistical Yearbooks, provincial statistical yearbooks, China Health Statistical Yearbooks, China Education Statistical Yearbooks, China Commerce Yearbooks, CNNIC statistical reports on internet development, household consumption statistics, food price statistics, and available child health or student physical fitness monitoring reports. Direct links to the main public data sources are provided below: National Bureau of Statistics of China/China Statistical Yearbooks: https://www.stats.gov.cn/english/Statisticaldata/ China Statistical Yearbook 2025: https://www.stats.gov.cn/sj/ndsj/2025/indexeh.htm CNNIC Statistical Reports: https://www.cnnic.com.cn/IDR/ReportDownloads/ Ministry of Education of China – Educational Statistics: https://en.moe.gov.cn/documents/statistics/ National Health Commission of the People’s Republic of China: https://en.nhc.gov.cn/ The processed province-year analytical dataset and variable construction details are available from the corresponding author upon reasonable request. No repository accession number is applicable.
